# Is degradation of dyes even possible without using photocatalysts? – a detailed comparative study†

**DOI:** 10.1039/d2ra05779d

**Published:** 2022-11-30

**Authors:** Subhadeep Sen, Chanchal Das, Narendra Nath Ghosh, Nabajyoti Baildya, Sumantra Bhattacharya, Moonis Ali Khan, Mika Sillanpää, Goutam Biswas

**Affiliations:** Department of Chemistry, Cooch Behar Panchanan Barma University Cooch Behar West Bengal 736101 India goutam@cbpbu.ac.in; Department of Chemistry, Pakuahat A. N. M. High School Malda India 732138; Department of Chemistry, Milki High School Milki Malda West Bengal India 732209; Department of Chemistry, National Institute of Technology Sikkim. Barfung Block Ravangla South Sikkim Sikkim 737139 India; Chemistry Department, College of Science, King Saud University Riyadh 11451 Saudi Arabia; Department of Biological and Chemical Engineering, Aarhus University Aarhus C Denmark; Department of Chemical Engineering, School of Mining, Metallurgy and Chemical Engineering, University of Johannesburg P. O. Box 17011, Doornfontein 2028 South Africa

## Abstract

Herein, catalyst-free, eco-friendly, photo-triggered, self-degradation of malachite green (MG) and crystal violet (CV) dyes in comparison to photocatalytic degradation were investigated. To the best of our knowledge, this is the first systematic study to demonstrate the reactive oxygen species (ROS), electron (e^−^) and hole (h^+^) generation ability of dyes to initiate self-degradation in the presence of direct solar energy (a free source of UV radiation) and UV light (254 and 365 nm). Various experimental conditions, *e.g.*, different dye concentrations, pH, vessel-materials (borosilicate glass and quartz) were optimized to achieve the optimum degradation outcomes. The degradation kinetics of dyes suggested the applicability of second-order-kinetics to all kinds of applied light sources. Investigation of the thermodynamic approach reveals that the self-degradation procedure was endothermic, with activation energies of 46.89 and 52.96 kJ mol^−1^, respectively, for MG and CV. The self-degradation mechanism was further corroborated by the quantum calculations, while the formation of final degraded products for dye-degradations was established on the basis of mass spectroscopy and total organic carbon (TOC) analysis. The computed emission energies for MG and CV advocate that the excitation energy occurs due to the sole-attribution electron excitation from the Highest Occupied Molecular Orbital (HOMO) to the Lowest Unoccupied Molecular Orbital (LUMO). The close energy difference between the hydroxyl anions and the dyes also facilitates the creation of the hydroxyl radical. In a similar manner, the excited electrons from the aforementioned dyes may readily be transferred to triplet molecular oxygen, which makes it possible to generate super oxide. The radical generated in the process facilitates the self-degradation of the dyes.

## Abstract 2

Degradation of cationic dyes through self generation of ROS.

## Graphical abstract

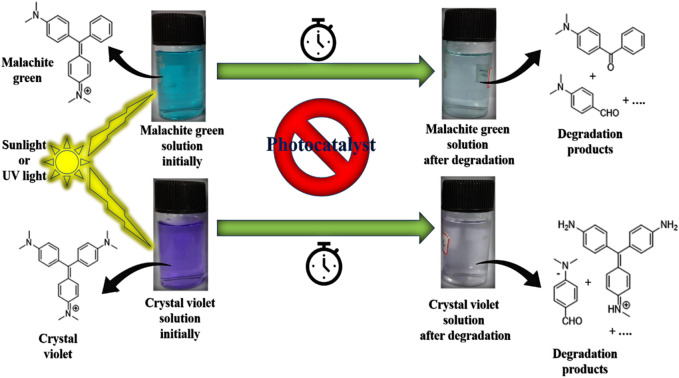

## 1. Introduction

Malachite green (MG) and crystal violet (CV) are the two most common dyes used for a variety of essential applications. CV is utilized in the textile industry and also as a biological strain.^1,2^ However, studies have reported CV as a mutagenic, cytogenic, and carcinogenic agent. It can exert a hazardous impact on human *viz.* digestive tract irritation, skin irritation, respiratory, and kidney failure, *etc.* In addition, it is a potent carcinogen and clastogen towards aquatic animals, and can promote tumorous growths in various fish species. Adverse effects of CV also include the reduction of photosynthetic activity of aqua-plants, inhibition of germination of seeds, and the ability to affect the microbes which are responsible for the biochemical nitrogen-cycle making the soil unproductive.^1^ MG, on the other hand, is used as a fungicide to prevent fungal growth in fish eggs, as an antibacterial agent, and in a variety of other applications.^3^ Even though this dye is widely used in aquaculture and the dye industry, it has limited use because, it persists in the eco-system and thus harms the receiving aquatic environment.^4^ Mutagenesis, carcinogenesis, teratogenicity, chromosomal breakage, and reduced fertility are also reported in aquatic species after a certain level of MG exposure. It can also harm mammals; for example, in rats, it causes tumors in lungs, breasts, ovary, and liver, as well as spleen, kidney, heart. MG is genotoxic, can damage the reproductive and immune systems, as well as found to be carcinogenic to the liver, thyroid and other organs of experimental mammals and impart cytotoxicity to mammalian cells.^3^ Hence, these dyes must be removed from the industrial wastewater before they discharged to water bodies.

Treatment of these waste effluents through photocatalytic degradation and adsorption processes by employing nanoparticles, and polymeric materials is gaining considerable attention.^5–12^ The other commonly used treatment processes include reverse osmosis, electrocoagulation, filtration, evaporation, membrane separation, and chemical precipitation. However, operational complexity, capital investment, and time consumed are the major hurdles towards restricting their wide-range application. When compared to other procedures, degrading dye contaminants in aqueous systems using solar irradiation in the presence of a photocatalyst is a straightforward and effective pathway.^13–17^ Additionally, sunlight is an ecologically benign and reliable source of energy that may be used for a wide range of purposes. The key advantage of solar irradiation during wastewater treatment is that it avoids secondary waste generation, thus making the treated water readily useable.

In recent times, UV rays (200–380 nm) have been widely used for the purification of wastewater by killing germs (like harmful bacteria including *Escherichia coli*, *Staphylococcus aureus*, *etc.*). Welch *et al.* (2018) experimentally proved that far UV-C rays (207–222 nm) are more efficient towards the killing of aerosolized H1N1 influenza virus without affecting normal human cells. Therefore, direct sunlight and UV rays (254 and 365 nm) are used for simultaneous degradation of harmful dyes and to kill germs in the wastewater.^18^

Previously, few works have reported about the self-degradation process of dyes. Though, the mechanistic pathway was not established clearly.^19,20^ Chiu *et al.* reported the generation of reactive species in semiconductor photocatalysts, which can directly degrade dye molecules,^16^ and also predicted the possibilities of self-degradation of dyes based on the position of conduction band and LUMO level of dye molecules. Thus, the goal of this study was to look into the feasibility of generating reactive species in dye without the presence of a semiconductor or photocatalyst, which would allow for self-degradation. To the best of our knowledge, this is the first time that an eco-friendly and economically appealing photo-assisted degradation of MG and CV dyes is established in a systematic method (with comparison), using variable experimental conditions. The kinetics of dye degradation by direct sunlight, along with UV rays of wavelength 365 nm (UV-365), and 254 nm (UV-254), were also measured. The generation of the ROS, e^−^ and h^+^ was investigated. Apart from that, activation energies (*E*_act_) for the self-degradation of the dyes were calculated from the Arrhenius equation, followed by other thermodynamic parameters, *e.g.*, change in Gibb's free energy, entropy, and enthalpy. The self-degradation of dyes by sunlight or UV light was confirmed from products detected by mass spectroscopy and TOC analysis.

## 2. Materials and methods

### 2.1. Chemicals and reagents

Two organic cationic dyes, CV (ACS reagent, ≥90.0% anhydrous basis) solid, and MG 1% (w/v) solution, were used in the entire experiment and were all purchased from Sigma-Aldrich, India. Iron oxide nanoparticles (Fe_3_O_4_) and titanium oxide (TiO_2_) nanoparticles were procured from SRL chemicals, India. UV-365 (UV-A) and UV-254 (UV-C) light sources were two different tube lights of 8 W each, purchased from the Phillips company. All the solutions were prepared in high purity deionized (D.I) water collected from Milli-Q water system. All chemicals were used without further purification.

### 2.2. Dye degradation experiment

The degradation of MG and CV dyes was performed at room temperature in a 100 mL borosilicate Erlenmeyer flask with UV-365 (intensity at 0.15 m: 800 μW cm^−2^), UV-254 (intensity at 0.15 m: 820 μW cm^−2^), and sunlight (Solar flux = 1370 W m^−2^),^21^ (24^th^ June, 25^th^ June, 13^th^ July, 6^th^ November, 9^th^ November 2021; latitude and longitude: 26°19′17.5368′′ N and 89°28′10.4412′′ E). The samples were collected at specified time intervals and the absorbance of the dyes was recorded using a UV-vis spectrophotometer at the maximum absorption wavelength of the respective dyes, *i.e.*, 618 nm for MG and 590 nm for CV. The experimental parameters such as time (0–300 min), initial concentration of dyes (2–8 mg L^−1^), pH of the solution (∼4–10), temperature (293, 303, and 313 K), and the material of the flask (borosilicate glass and quartz) were varied during the experiment.

The pH of the solution was adjusted with 0.1 M hydrochloric acid (HCl) and 0.1 M sodium hydroxide (NaOH) solutions and was determined by a pH meter (Fisher Scientific Accumet). For the investigation of the mechanism, different scavengers (*e.g.*, methanol, isopropanol, and ethylenediaminetetraacetic acid disodium salt) were used.

For better consistency, all experiments were performed in duplicate. If the data was inconsistent, the experiments were repeated. The percentage degradation of the dyes was estimated using the following equation [eqn (1)]:1
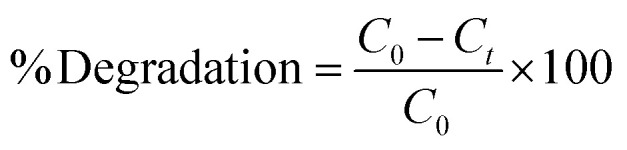
where *C*_0_ and *C*_*t*_ represent the concentration of the dye before and after a certain irradiation time, respectively.

### 2.3 Analytical analysis

The required amount of dye was kept in a 100 mL borosilicate Erlenmeyer flask (Remco India), 0.2 m away from the respective UV lamp in a closed UV chamber. The UV-vis measurement of the solution was performed by Thermo Scientific Evolution 201 UV-vis spectrophotometer using a 0.01 m path length quartz cuvette (purchased from the TUV company). The mass spectra were obtained by the ES-MS system (Waters Micromass QTof MicroTM).

### 2.4. Computational analysis

All the simulations in the present work have been conducted employing density functional theory (DFT) using the Gaussian 16 program.^22^ Ground state geometries of MG and CV dyes were optimized using B3LYP/6-31+G (d,p) level of theory. During optimization the effect of solvent (water) was taken into account by Integral Equation Formalism of Polarizable Continuum Model (IEFPCM) as implemented in Gaussian program.^23^ Additionally, an ultrafine integral grid-based calculation scheme was also considered while performing all calculations to reduce the numerical error during calculation. Using the same level of theory, the vibrational frequency calculation of the molecules was performed and no imaginary frequency clearly signifies that the optimized geometry resides to the minima on the potential energy surface. Following the optimized ground state geometry, time-dependent DFT (TD-DFT) calculations were performed to obtain excited state properties by accounting lowest 30 singlet excitations. In our TD-DFT calculations, solvent effects (water) were incorporated by applying the Polarizable Continuum Model (PCM) using the integral equation formalism variant.^23^ To the best of our knowledge there is no report for the TDDFT calculation of these dyes, hence to calibrate our TDDFT data with the experimental one different hybrid functionals *viz.* B3LYP,^24^ PBEPBE in conjunction with the 6-31+G(d,p) basis sets were chosen and analyze the effect of different functional on the excitations.^25^ The corresponding TDDFT data were collected in Table S1,† which revealed that the PBEPBE hybrid functional reproduces the experimental data with reasonably high accuracy. To visualize the nature of transition states, GaussSum 3.0.2 package was employed for evaluating the major orbital contributions.^26^

## 3. Results and discussion

### 3.1. Effect of initial dye concentration and light sources

**Fig. 1:**
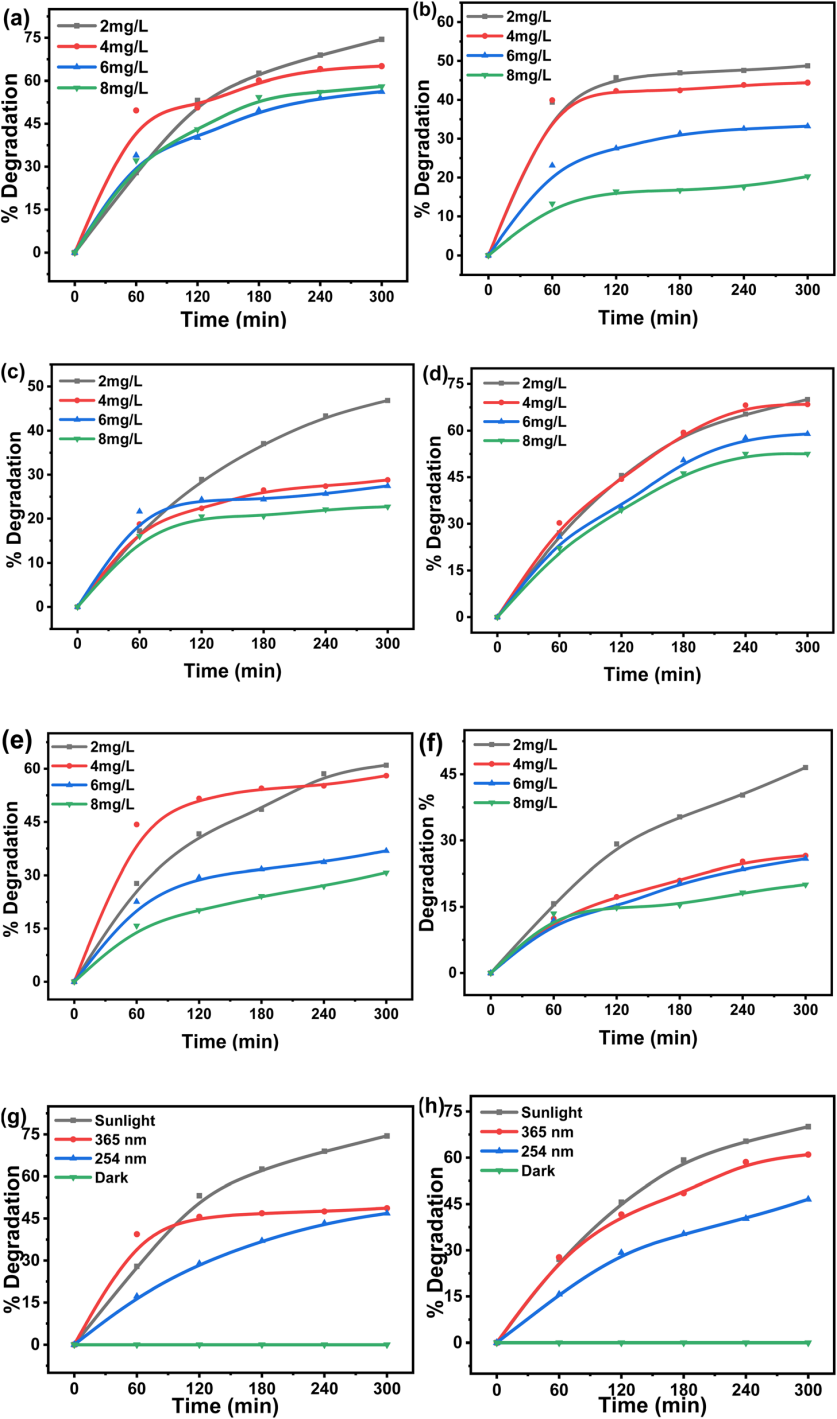
Degradation of MG in the presence of (a) sunlight, (b) UV-365, (c) UV-254 and for CV in the presence of (d) sunlight, (e) UV-365, (f) UV-254 at different initial concentrations; comparison of (g) MG and (h) CV degradation at different conditions.

Two dyes were separately tested to find % degradation at various solution concentrations (2, 4, 6, and 8 mg L^−1^) for 300 min in three different light sources: UV-365, UV-254, and direct sunlight. The results displayed in Fig. 1(a)–(f) reveal that with an increase in initial dye concentration, the degradation of both dyes under three different light sources was reduced considerably. Both MG and CV at 2 mg L^−1^ initial concentration exhibited maximum degradation of 74 and 70% under sunlight, followed by 48 and 61% under UV-365 and 47 and 46% under UV-254, respectively. This was because, with an increase in dye concentration, a greater amount of light gets absorbed in solution, resulting in a small amount of light being utilized for the photocatalytic process.^27^ Also, higher initial dye concentrations dampen the photocatalytic reaction, by shortening the path length of the photon entering the solution.^28^ Therefore, the production of charge carriers and reactive radicals was simultaneously reduced. This results in a reduction in photodegradation.^16^ Hence, the dyes' degradation was inversely proportional to their concentration. In this experiment, 2 mg L^−1^ concentration of both MG and CV exhibited the maximum degradation. All subsequent experiments (effect of pH, material of the Erlenmeyer flask, temperature, and the presence of scavenger) were performed at this concentration.

The greater percentage of dye degradation under sunlight was probably due to the presence of a wide ranges of wavelengths in comparison to fixed UV-irradiation (UV-365 and UV-254). Surprisingly, the percentage of dye degradation was comparatively better with UV-365 than with UV-254 irradiation, though the latter consists of higher energy. This might be because of the borosilicate material of the flask, which has a cutoff at around 300 nm. Hence, only a limited amount of UV-254 can penetrate, thus, lowering the degradation.^29^ Additionally, it can be proposed that the separation of photoexcited electrons and holes competes with their recombination at low light intensities. This prevents the formation of reactive radicals. As the light intensity increases, electron–hole formation becomes prominent, resulting in a rapid degradation rate.^16^ For the negative control experiment, both dyes (concentration of 2 mg L^−1^ each) were kept in the dark and the outcome (Fig. 1g and h) exhibited that the degradation was negligible.

### 3.2. Kinetics study

The dye degradation kinetics were studied using the batch method. Each dye solution (10 mL, 2 mg L^−1^) was taken in a borosilicate Erlenmeyer flask, and then exposed to UV-365, UV-254, and direct sunlight, respectively, for 300 min. The absorbance was measured at every 60 min interval.

A kinetics study was evaluated by using the first [eqn (2)], second [eqn (3)], and zero [eqn (4)] order kinetic models of the linear type.

2 First-order-equation: ln *C*_*t*_ = −*k*_1_*t* + ln *C*_0_

3
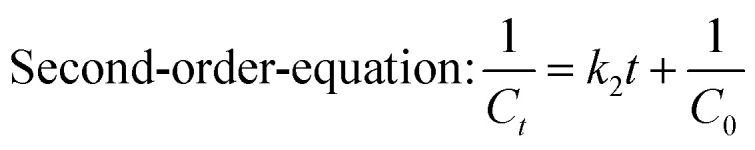

4 Zero-order-equation: *C*_*t*_ = −*k*_0_*t* + *C*_0_

*C*_0_ and *C*_*t*_ are the initial concentration and concentration after time *t*; *k*_1_, *k*_2_, and *k*_0_ are the rate constants of first-order, second-order, and zero-order equations, respectively. Graphs of ln *C*_*t*_*versus t*, 1/*C*_*t*_*versus t*, and *C*_*t*_*versus t* were plotted to check whether the degradation of respective dyes obeys first, second, or zero-order kinetics.^30^

**Fig. 2:**
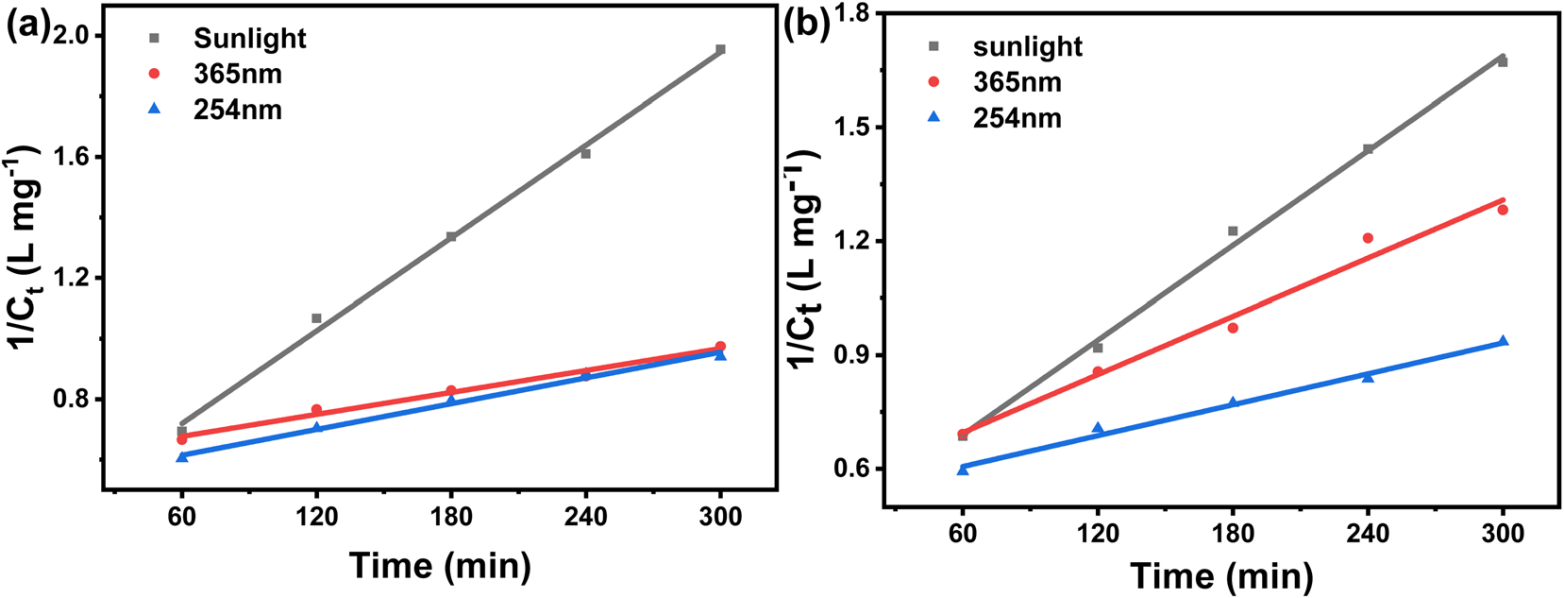
Second order kinetic plots of (a) MG and (b) CV under sunlight, 365 nm, and 254 nm light.

The data obtained from the previous experiments using, first-, second-, and zero-order equations [eqn (2)–(4)], are tabulated in Table 1, along with the rate constants and correlation coefficient (*R*^2^) values. The correlation coefficient (*R*^2^ = 0.9965 for MG and *R*^2^ = 0.9966 for CV) and the comparison of the calculated initial concentration (*C*_0,cal_) with the actual initial concentration (*C*_0,ac_) confirmed all of the experimental data. The tabulated data (Table 1) suggests that the second-order kinetic model fits well with the kinetic data over the entire contact time range for both MG and CV under UV-365 as well as UV-254, because the *R*^2^ value is near to unity [eqn (2a), (2b)] (Fig. 2).

**Table 1:** Kinetic parameters MG and CV degradation^a^

Light source	Dye	Zero-order			First-order			Second-order		
		*C*_0 (cal.)_ (mg L^−1^)	*R* ^2^	*k*_0_ (mg L^−1^ min^−1^)	*C*_0 (cal.)_ (mg L^−1^)	*R* ^2^	*k*_1_ (min^−1^)	*C*_0 (cal.)_ (mg L^−1^)	*R* ^2^	*k*_2_ (L mg^−1^ min^−1^)
Sunlight	MG	1.5046	0.8853	0.0036	1.8578	0.9633	0.0041	2.5063	0.9965	0.0051
	CV	1.5652	0.9255	0.0035	1.7329	0.9741	0.0037	2.2904	0.9966	0.0042
UV365 nm	MG	1.5702	0.9618	0.0019	2.9661	0.977	0.0015	1.6548	0.9839	0.0012
	CV	1.5514	0.953	0.0028	3.1361	0.9748	0.0026	1.8439	0.9815	0.0026
UV254 nm	MG	1.7486	0.9576	0.0025	1.8011	0.9785	0.0019	1.8896	0.992	0.0014
	CV	1.7684	0.9544	0.0024	1.8198	0.9773	0.0018	1.9069	0.9893	0.0014

a Initial dyes concentration (*C*_0_): 2 mg L^−1^.

### 3.3. Effect of pH

**Fig. 3:**
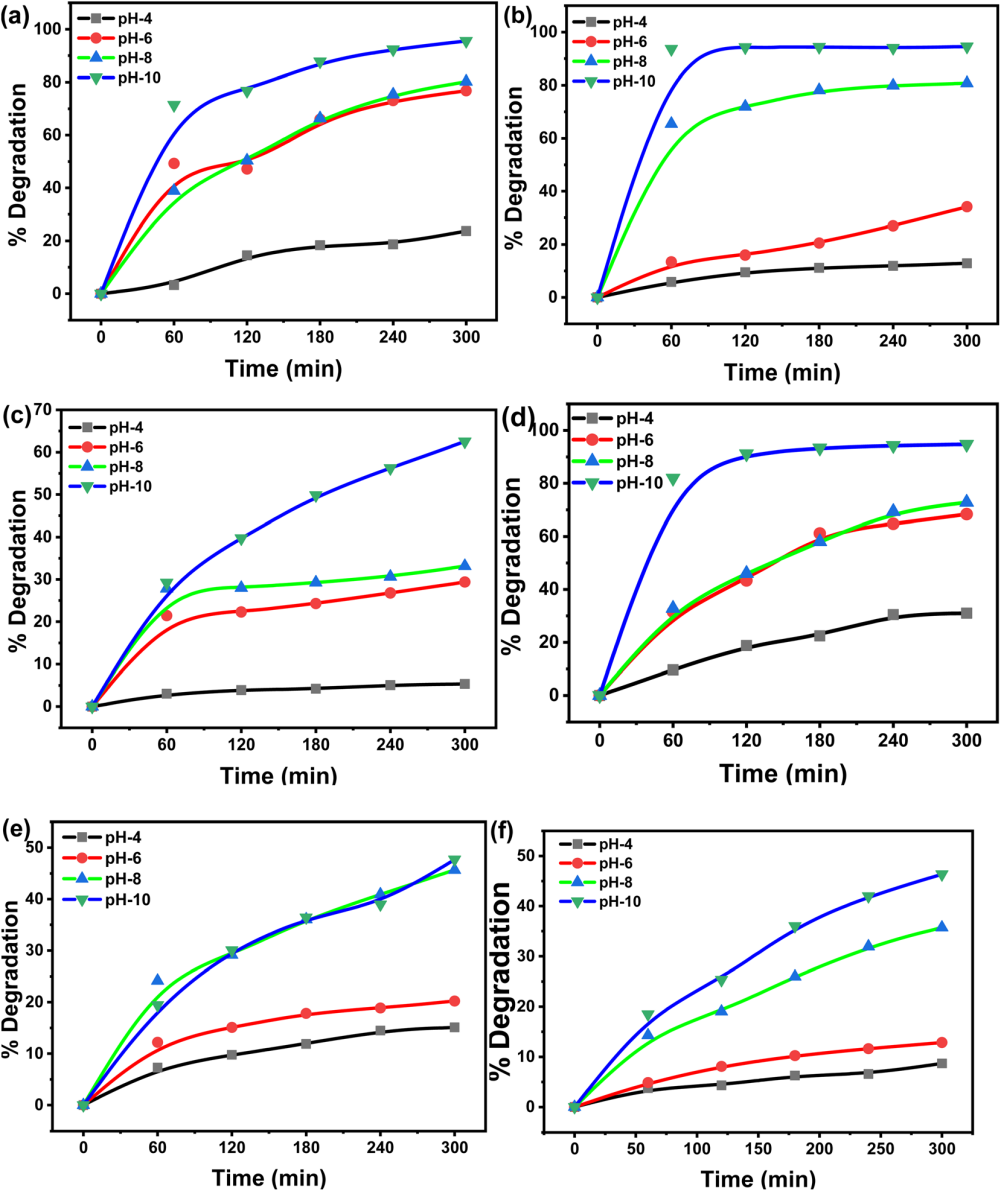
Degradation of MG under (a) sunlight, (b) UV-365, (c) UV-254 and of CV under (d) sunlight, (e) UV-365, (f) UV-254 at different pH.

The pH of dye solutions has a major impact on the degradation process depending on their types (cationic, anionic, or neutral). However, evaluating the influence of pH on the degradation process is a challenging task. This is because three distinct chemical routes may contribute to dye degradation, such as hydroxyl radical attack, direct oxidation by the positive hole, and direct reduction by the electron in the conducting band. Each contribution is governed by the type of substrate and its pH. The effect of solution pH (between 4 and 10) on MG and CV degradation were investigated and found to have a major impact. Fig. 3(a)–(f) illustrates MG and CV degradation over a fixed time at varied solution pH. During the study, it was observed that with an increase in pH, the dye degradation increases. The maximum degradation occurred at pH ∼ 10, and the respective degradation percentages of MG and CV were 96 and 95% under sunlight, followed by 94 and 47% under UV-365, and 62 and 46% under UV-254 irradiations. For MG, this could be owing to the fact that hydroxyl radicals (˙OH) are predominantly responsible for the oxidation process at higher pH ranges. Photodegradation decreased in acidic media due to a decrease in ˙OH but increased in alkaline media due to ˙OH release. Again, CV contains two acid dissociation constants (p*K*_a1_ 5.31 and p*K*_a2_ 8.64), therefore, decolorization should be more efficient at higher doses. As a result, at alkaline pH, a higher removal of CV was expected, which correlates with dissociation constants. In addition, the cationic nature of CV encourages decolorization at higher pH values.^31^

### 3.4. Effect of flask materials

**Fig. 4:**
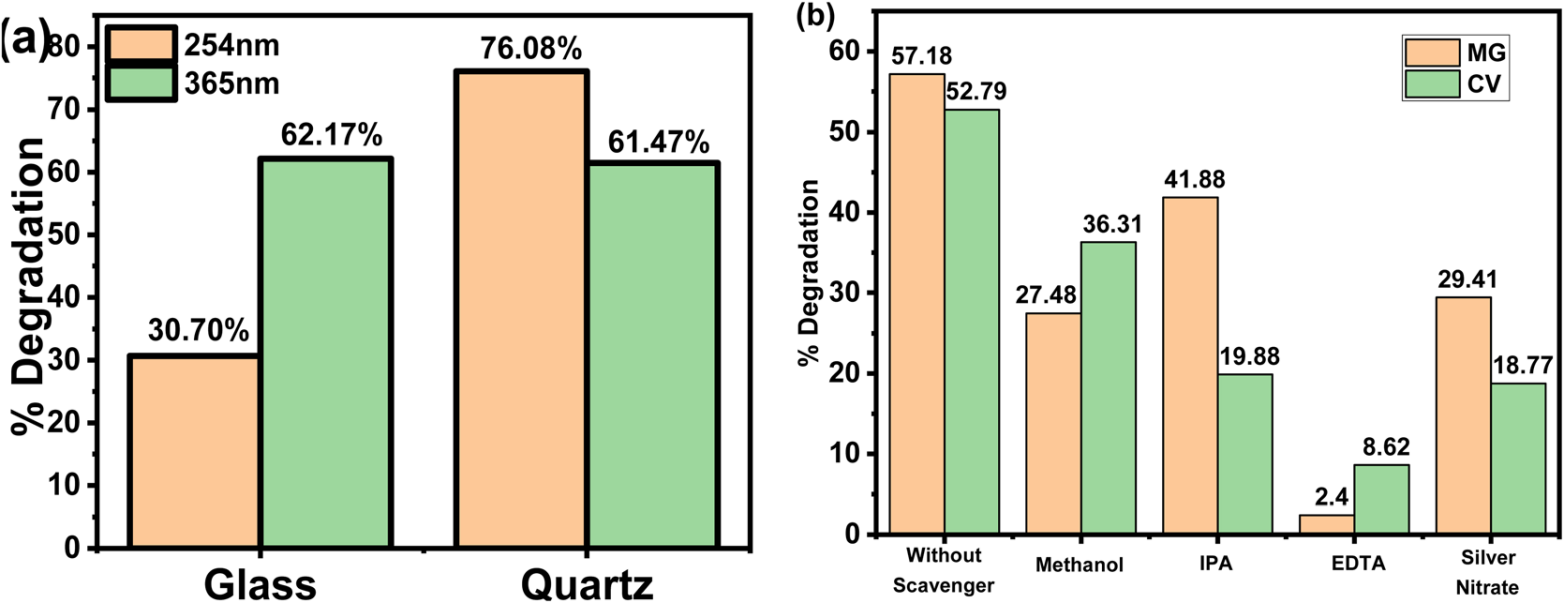
(a) Percentage of degradation of MG through different surfaces under UV-365 and UV-254. (b) Trapping experiment of active species during the photocatalytic degradation of MG and CV under UV-365 irradiation.

To investigate the effect of flask material on the degradation reaction, 2 mg L^−1^ of MG (3 mL) were placed in two separate 0.01 m path length cuvettes made up of borosilicate glass and quartz and were exposed to different UV light sources for 300 min. Fig. 4(a) shows that with UV-365, there was no significant variation in MG degradation between borosilicate glass and quartz. This was probably because of the larger wavelength (*e.g.*, here 365 nm) passed through both glass and quartz with no substantial absorption. An intriguing result was observed under UV-254 with a substantial decrease in MG degradation from 76% in quartz to just 30% in glass cuvette. As discussed before, this was due to partial permeation of UV irradiation through borosilicate glass at 254 nm; as a large amount of the shorter wavelength was absorbed.^29^ Consequently, the transmission of UV irradiation was reduced in the dye solution, resulting in a lower degradation percentage. In contrast, the quartz cuvette does not absorb any 254 nm irradiation. Hence, the MG degradation increases compared to at 365 nm, probably due to higher energy content.^32^

### 3.5. Effect of scavenger

To better understand the mechanism of photocatalytic process, reactive species trapping study was performed in the presence of several radical quenchers at natural pH. Quenchers are the chemical compounds that prevent certain specific species from participating in the degradation reaction by trapping them during the photocatalytic experiment. Methanol (MeOH),^33^ isopropanol (IPA),^33^ silver nitrate^16^ and ethylenediaminetetraacetic acid disodium salt (EDTA-Na_2_)^34^ were used as ˙O_2_^−^, ˙OH, e^−^ and h^+^ scavengers, respectively. The control experiment was conducted under similar conditions without any quenchers. According to Fig. 4(b), the presence of quenchers significantly reduced photocatalytic degradation. The degradation efficiency in the presence of 1 mmol of MeOH was observed to be modestly reduced for both dyes. This observation concluded the participation of (˙O_2_^−^) radical species in the degradation mechanism. Therefore, the addition of MeOH quenches (˙O_2_^−^) radical formation, thus resulting in decreased degradation. Similarly, the addition of 1 mmol of IPA within the dye solution results in a considerable reduction in degradation, confirming the participation of the ˙OH radical in the process. The electron (e^−^) had a significant effect in the degradation process, which was confirmed by the quenching experiment using silver nitrate, which reduced the degradation to 29.41 and 18.77% for MG and CV respectively. When 1 mmol of EDTA-Na_2_ was used, the degradation of MG decreased from 57.18 to 2.40% and CV from 52.79 to 8.62%. This indicates that active species of h^+^ play a substantial role in the degradation mechanism. The findings showed that all three species play an active role in photocatalytic degradation. Therefore, it is possible to deduce that the h^+^ produced in the photocatalytic system is responsible for the improved photo-oxidation performance towards the decomposition process.

### 3.6. Effects of temperature on self-degradation of MG and CV

The Arrhenius relationship for determination of activation energy (*E*_act_) can be written in linear from eqn (5).5
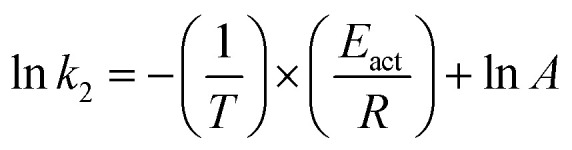
where, A, *E*_act_, *R*, and *T* represent the frequency factor (s^−1^), activation energy (kJ mol^−1^), universal gas constant (J mol^−1^ K^−1^), and absolute temperature (K), respectively.

The thermodynamic relation for the enthalpy of the reaction and the activation energy is given by eqn (6).^35^

6 *H*_A_ = *E*_act_ − *RT*

*H*_A_ is the enthalpy of activation.

The relation between the reaction rate constant, (*k*_2_) with the enthalpy (Δ*H*) and entropy (Δ*S*) is given by the Eyring equation (eqn (7)).7
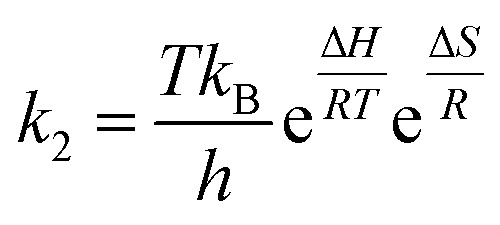
where *k*_B_ is the Boltzman's constant = 1.381 × 10^−23^ J^−1^ K^−1^, *h* is the Planck's constant = 6.626 × 10^−34^ J s.

The eqn (7) can be written as,8
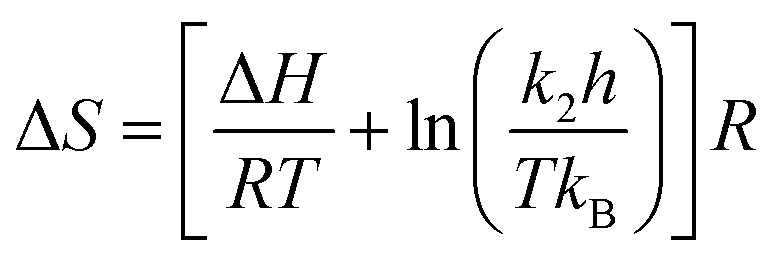


From the eqn (8) the entropy (Δ*S*) can be calculated with the help of above eqn (5)–(7).

Gibbs free energy (Δ*G*) can be calculated using eqn (9) which is the thermodynamic relation for the entropy, enthalpy and Gibbs free energy (Δ*G*)

9 Δ*G* = Δ*H* − *T*Δ*S*

The effect of temperature on the rate of MG and CV degradation was studied at natural pH under UV-365 at temperatures of 293, 303, and 313 K. At a regular time interval of 60 min, the UV-vis absorbance for degrading dye solutions was measured up to 300 min. The previously observed second-order rate constant (*k*_2_) was calculated by eqn (3) and, according to the Arrhenius equation [eqn (5), linear form], plotting was performed for ln *k*_2_ against 1/*T*. *A* and *E*_act_ were calculated from the intercept and slope, respectively.

**Fig. 5:**
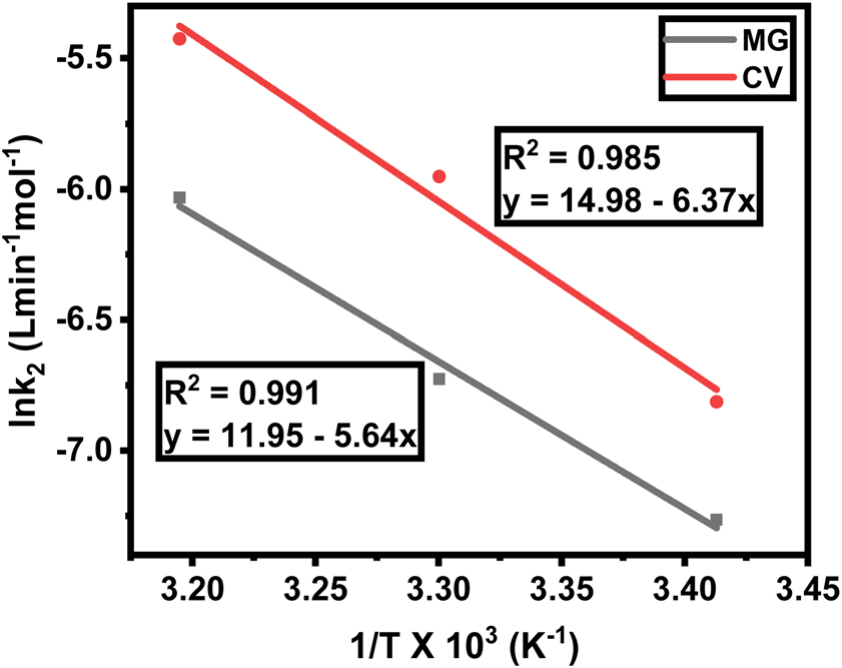
Temperature dependency of rate of degradation, plot of ln *k*_2_ (L min^−1^ mol^−1^) against 1/*T* (K^−1^) for MG and CV under UV-365.

While checking the Arrhenius behavior, the *k*_2_ values were ranged as 0.7 × 10^−3^ to 2.4 × 10^−3^ L mol^−1^ min^−1^ for MG and 1.1 × 10^−3^ to 4.4 × 10^−3^ L mol^−1^ min^−1^ for CV, respectively, in the temperature range between 293 and 313 K. In each case, there were linear plots with *A* = 1.55 × 10^5^ s^−1^ (for MG) and 3.20 × 10^6^ s^−1^ (for CV) and *E*_act_ = 46.89 kJ mol^−1^ (for MG) and 52.96 kJ mol^−1^ (for CV), respectively. The respective *R*^2^ values were 0.991 and 0.985 for MG and CV. It was also observed from Table 2 that *k*_2_ increases approximately twice for every ten degrees rise in temperature (Fig. 5). Hence, both MG and CV obey the Arrhenius equation [eqn (5)]. Now, it should be noted that for both dyes, the free energy change (Δ*G*) was positive (Table 2) and hence the degradation reactions were non-spontaneous. So, to initiate the reaction, some sort of energy must require.^35^

**Table 2:** Arrhenius parameters for the degradation of MG and CV under UV-365

Dye	*T* (K)	*k*_2_ (L min^−1^ mol^−1^)	*A* (s^−1^)	*E*_act_ (kJ mol^−1^)	*R* ^2^	Δ*H* (kJ mol^−1^)	Δ*S* (kJ mol^−1^ K^−1^)	Δ*G* (kJ mol^−1^)
MG	293	0.7 × 10^−3^	1.55 × 10^5^	46.89	0.991	44.45	−0.153	89.41
	303	1.2 × 10^−3^				44.37	−0.154	91.10
	313	2.4 × 10^−3^				44.28	−0.153	92.30
CV	293	1.1 × 10^−3^	3.20 × 10^6^	52.96	0.985	50.52	−0.128	88.31
	303	2.6 × 10^−3^				50.44	−0.127	89.15
	313	4.4 × 10^−3^				50.35	−0.129	90.73

### 3.7. Computational analysis

**Fig. 6:**
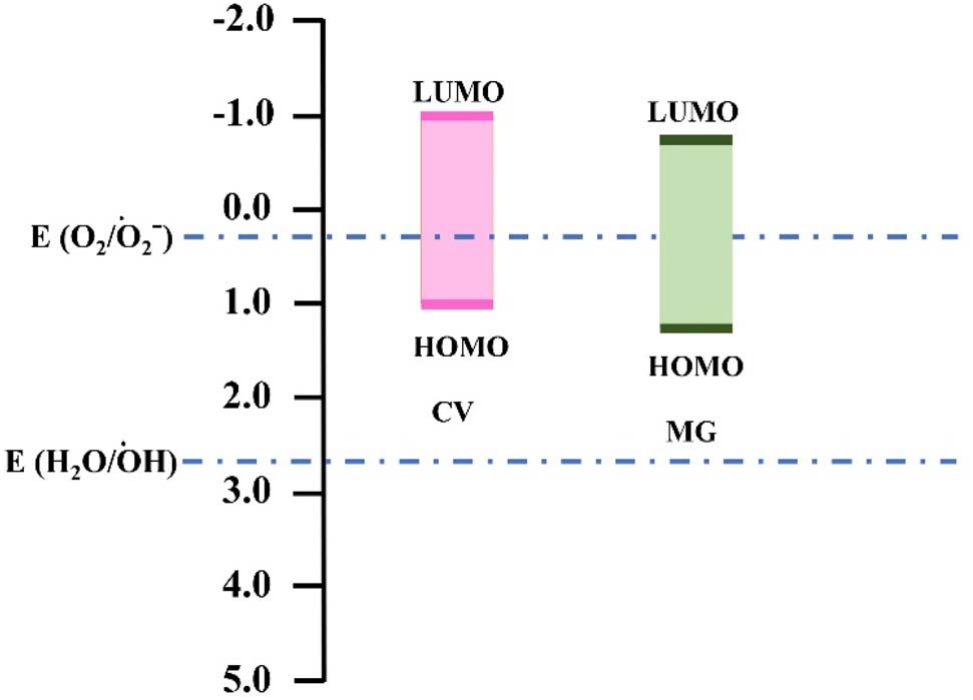
Redox potentials for the radical generation and HOMO, LUMO energy alignment for the dyes.

To gain insight on the mechanism of self-degradation of dyes we have performed DFT calculations. Previous studies reported that the HOMO, LUMO energy levels of these dyes are aligned suitably for O_2_/O_2_˙^−^ radical generation as illustrated in the Fig. 6.^16^ On the other hand, our theoretical calculation indicates that the HOMO and LUMO energy levels for CV and MG were −5.72 eV, −3.04 eV and −5.82 eV, −3.29 eV respectively. The band gap for CV and MG were 2.68 eV and 2.53 eV, which falls in the range of visible excitation energy of 1.8–3.2 eV. This confirms that visible light driven photoexcitation, is possible for both of the dyes.^36^

We have also analyzed the major orbital contribution in the electronic excitation along with oscillator strengths of these dyes and recorded in the Table S2.† We find that at wavelength maxima (*λ*_max_) *i.e.*, at 573 nm for CV and 570 nm for MG the corresponding major transitions are H-1 → L (46%), H → L (54%) and H → L (100%) respectively. This data indicates that visible light driven photoexcitation can lead to the generation of excited electron, hole pair which can initiate the radical generation (O_2_/O_2_˙^−^) and starts the degradation process. The involvement of electron, hole pair in the degradation process has also been confirmed by our radical scavenging experiments.

For excitation of electrons from Valence Band (VB) (equivalent to HOMO) to Conduction Band (CB) (Equivalent to LUMO), the position of HOMO and LUMO for both MG and CV dyes should be located.^37,38^ Comparison of the computational data for energy levels of CB and VB with the previously reported cyclic voltammetry data, it was found that the computational calculations were in good agreement with the previously reported cyclic voltametric analyzed data. According to Ng *et al.* the experimental band gap for CV and MG were 1.91 and 1.62 eV respectively.^39^ Comparison of computational data and reported data are tabulated in Table S3.†

### 3.8. Mechanism

**Fig. 7:**
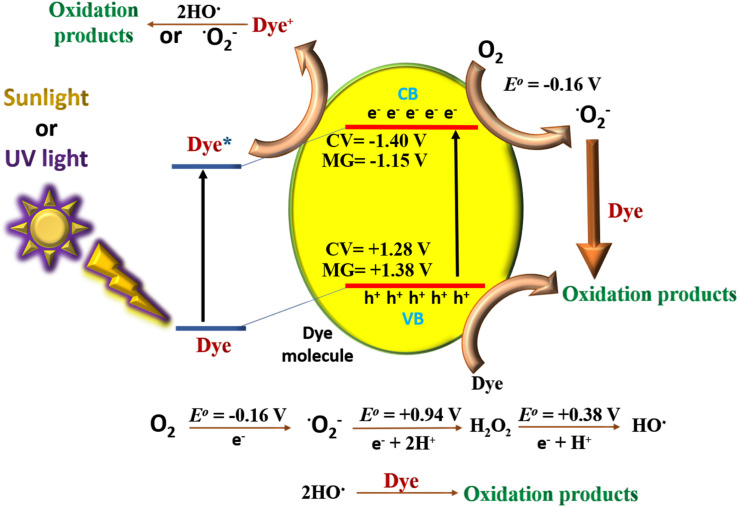
Schematic illustration of dyes degradation pathway.

**Fig. 8:**
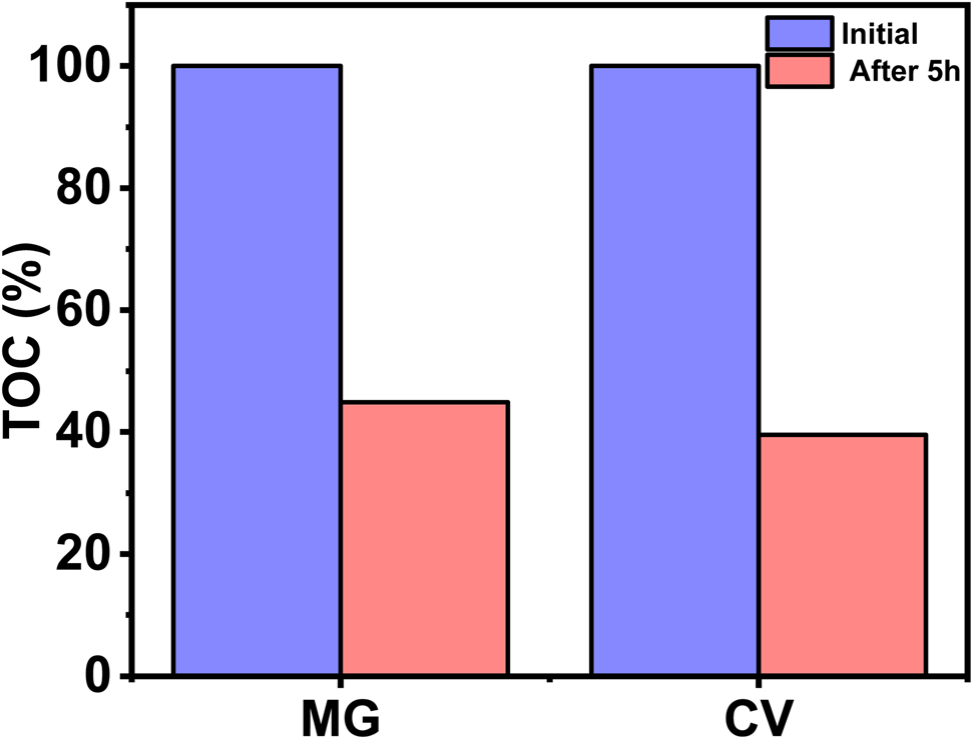
Reduction of TOC (%) for CV and MG dyes (2 mg L^−1^), before and after degradation in sunlight after 5 h.

The plausible mechanism for the self-degradation of the dye in the presence of sunlight/UV rays is illustrated in Fig. 7. Initially the light energy was absorbed by the dye and moved to the excited state. Similarly, electrons from valence orbital (VO) moved to unoccupied orbital (UO) by absorbing photons of suitable energy. In this process of transition, OH˙, and ˙O_2_^−^ are generated, which further degrades the dye. In general, photocatalytic water oxidation is governed by a proton removal reaction which involves both the breaking of the O–H bond and the trapping of a photogenerated hole (H_2_O* + h^+^ → ˙OH* + H^+^) in the valence band, but as the HOMO of MG and CV lies in a higher position compared to the redox potential of H_2_O/OH˙, hence OH˙ generation occurs in an indirect manner only (from superoxide radical). Diffusion of ˙OH* species away from the surfaces allows the formation of free ˙OH radicals, which are important components in the removal of organic pollutants.^40,41^ In the mechanism, e^−^ and h^+^ have a major role to play. The involvement of radicals (ROS) is also supported by the scavenging experiment (Section 3.5). The degraded products were characterized by mass spectroscopy and the results were compared with the reported ones from the literature (Fig. S1†).^41–43^ The TOC analysis for the degradation of dyes were performed after 5 h irradiation with sunlight (Fig. 8). The final % of TOC for CV and MG were 39.54 and 44.87% respectively. This data confirmed the degradation of dyes by the irradiation of light.

### 3.9. Comparison with other photocatalyst

**Fig. 9:**
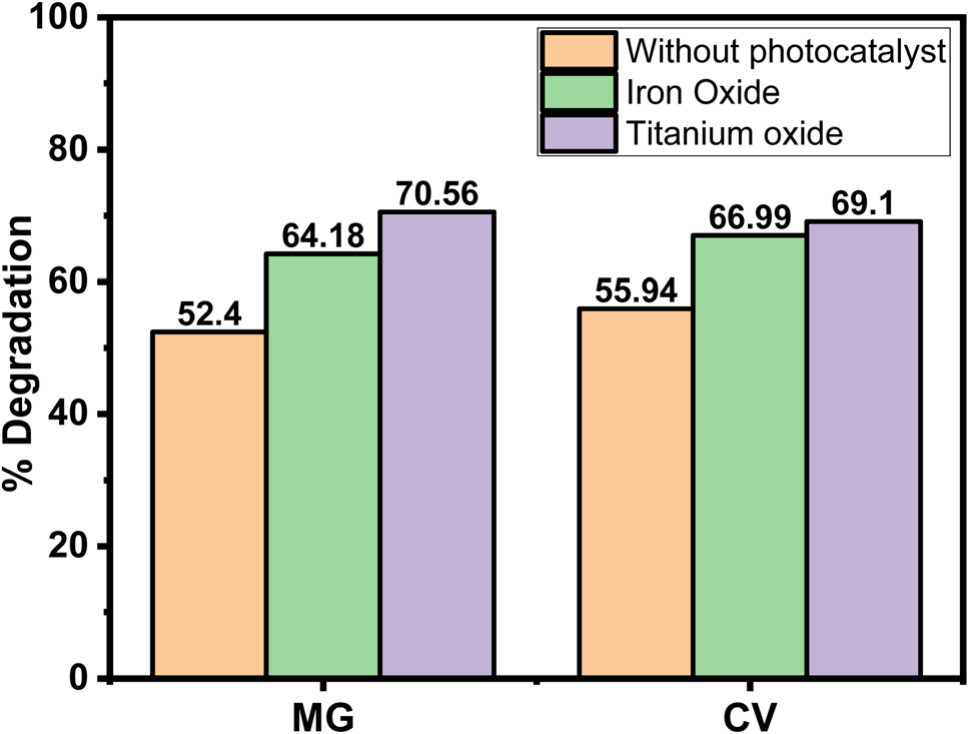
Percentage of degradation of MG and CV without any photocatalyst and in presence of Fe_3_O_4_ and TiO_2_ nanoparticles in 365 nm wavelength.

In order to understand the degradation efficacy in a better way, the degradation of the dyes was compared with some reported photocatalysts (positive control), *i.e.*, Fe_3_O_4_ and TiO_2_ nanoparticles at 365 nm wavelength. The results revealed that (Fig. 9) in the presence of both the photocatalyst the degradation efficacy was higher compared to the absence of any photocatalyst, which was expected. Both the Fe_3_O_4_ and TiO_2_ are reported to be an exceptional photocatalysts, having band gaps in the range between 2 and 3.5 eV.^44,45^ The most relevant finding was that the degradation percentage of the dye in the absence of a photocatalyst was comparable to that of a reported one.

## 4. Conclusions

In summary, an innovative approach for MG and CV degradation by irradiation of direct sunlight and UV rays of different wavelengths was established. The results provide a significant first step towards photocatalyst free degradation of organic dyes. The present findings might help to resolve toxicity or expense issues incurred during the purification of wastewater. In this study, it was observed that direct sunlight was most effective in the degradation of dyes compared to UV rays. As expected, high energy UV rays (UV-254) were more effective in degradation. However, due to absorbance by borosilicate glass, UV-365 rays exhibited more efficiency towards degradation. Also, at highly alkaline pH (pH = 10) the degradation was more effective in comparison with neutral or acidic pH. Second-order-kinetics was obeyed by both of the dyes, under all types of light sources. From the thermodynamical calculation, the *E*_act_ value was found to be 46.89 kJ mol^−1^ for MG and 52.96 kJ mol^−1^ for CV. The formation of ˙O_2_^−^, e^−^ and ˙OH radicals during the degradation process was also confirmed by using a suitable scavenger, which results in a deceleration of the rate of reaction. The theoretical calculation also corroborates their excitation process, which qualitatively suggests the energy transfer from the excited state to the ground state.

## Author contributions

Conceptualization, S. Sen, C. Das and G. Biswas; investigation and formal analysis, S. Sen, C. Das, N. Ghosh, N. Baidya and S. Bhattacharya; writing: original draft preparation, S. Sen, C. Das, N. Ghosh, S. Bhattacharya and G. Biswas; writing: review and editing, G. Biswas, N. Ghosh, S. Bhattacharya, Moonis Ali Khan and M. Sillanpää; supervision, G. Biswas; funding acquisition, G. Biswas. All authors have read and agreed to the published version of the manuscript.

## Conflicts of interest

The authors declare no competing financial interest.

## Supplementary Material

RA-012-D2RA05779D-s001

## References

[cit1] Mani S., Bharagava R. N. (2016). Exposure to Crystal Violet, its toxic, genotoxic and carcinogenic effects on environment and its degradation and detoxification for environmental safety. Rev. Environ. Contam. Toxicol..

[cit2] Kulkarni M. R., Revanth T., Acharya A., Bhat P. (2017). Resour.-Effic. Technol..

[cit3] Srivastava S., Sinha R., Roy D. (2004). Aquat. Toxicol..

[cit4] Bills T. D. , Marking L. L. and Chandler Jr J. H., Malachite green, its toxicity to aquatic organisms, persistence, and removal with activated carbon, 1977

[cit5] Das C., Sen S., Singh T., Ghosh T., Paul S. S., Kim T. W., Jeon S., Maiti D. K., Im J., Biswas G. (2020). Nanomaterials.

[cit6] Mudhoo A., Sillanpää M. (2021). Environ. Chem. Lett..

[cit7] Nazri M. K. H. M., Sapawe N. (2020). Mater. Today: Proc..

[cit8] Das C., Singh S., Bhakta S., Mishra P., Biswas G. (2021). Chemosphere.

[cit9] Wen W., Hai J., Yao J., Gu Y.-J., Kobayashi H., Tian H., Sun T., Chen Q., Yang P., Geng C., Wu J.-M. (2021). Chem. Mater..

[cit10] Wen W., Yao J.-C., Gu Y.-J., Sun T.-L., Tian H., Zhou Q.-L., Wu J.-M. (2017). Nanotechnology.

[cit11] Chen Y.-A., Wang Y.-T., Moon H. S., Yong K., Hsu Y.-J. (2021). RSC Adv..

[cit12] Ghosh N., Sen S., Biswas G., Singh L. R., Chakdar D., Haldar P. K. (2022). Int. J. Environ. Anal. Chem..

[cit13] Rajasulochana P., Preethy V. (2016). Resour.-Effic. Technol..

[cit14] Liu X., Song X., Zhang S., Wang M., Pan B. (2014). Phys. Chem. Chem. Phys..

[cit15] Wu J.-M., Wen W. (2010). Environ. Sci. Technol..

[cit16] Chiu Y.-H., Chang T.-F., Chen C.-Y., Sone M., Hsu Y.-J. (2019). Catalysts.

[cit17] Fang M.-J., Tsao C.-W., Hsu Y.-J. (2020). J. Phys. D: Appl. Phys..

[cit18] Welch D., Buonanno M., Grilj V., Shuryak I., Crickmore C., Bigelow A. W., Randers-Pehrson G., Johnson G. W., Brenner D. J. (2018). Sci. Rep..

[cit19] Sáenz-Trevizo A., Pizá-Ruiz P., Chávez-Flores D., Ogaz-Parada J., Amézaga-Madrid P., Vega-Ríos A., Miki-Yoshida M. (2019). J. Fluoresc..

[cit20] Fischer A. R., Werner P., Goss K.-U. (2011). Chemosphere.

[cit21] Solar Energy Flux – Solar Energy, https://solarenergycanada.org/solar-energy-flux/, accessed 2 November, 2022

[cit22] Citation|Gaussian.com, https://gaussian.com/citation/, accessed July 3, 2022

[cit23] Cossi M., Barone V., Cammi R., Tomasi J. (1996). Chem. Phys. Lett..

[cit24] Becke A. D. (1992). J. Chem. Phys..

[cit25] Perdew J. P., Burke K., Ernzerhof M. (1996). Phys. Rev. Lett..

[cit26] O’boyle N. M., Tenderholt A. L., Langner K. M. (2008). J. Comput. Chem..

[cit27] Reza K. M., Kurny A., Gulshan F. (2017). Appl. Water Sci..

[cit28] Saggioro E. M., Oliveira A. S., Pavesi T., Maia C. G., Ferreira L. F. V., Moreira J. C. (2011). Molecules.

[cit29] McMurray T. A., Byrne J. A., Dunlop P. S. M., McAdams E. T. (2005). J. Appl. Electrochem..

[cit30] Sidney Santana C., Nicodemos Ramos M. D., Vieira Velloso C. C., Aguiar A. (2019). Int. J. Environ. Res. Public Health.

[cit31] Abdi M., Balagabri M., Karimi H., Hossini H., Rastegar S. O. (2020). Appl. Water Sci..

[cit32] Bergamonti L., Alfieri I., Franzò M., Lorenzi A., Montenero A., Predieri G., Raganato M., Calia A., Lazzarini L., Bersani D., Lottici P. P. (2014). Environ. Sci. Pollut. Res..

[cit33] Dong S., Cui L., Zhao Y., Wu Y., Xia L., Su X., Zhang C., Wang D., Guo W., Sun J. (2019). Appl. Surf. Sci..

[cit34] Chen F., Yang Q., Li X., Zeng G., Wang D., Niu C., Zhao J., An H., Xie T., Deng Y. (2017). Appl. Catal., B.

[cit35] Salahudeen N., Rasheed A. A. (2020). Sci. Rep..

[cit36] Cestaro R., Schweizer P., Philippe L., Maeder X., Serrà A. (2022). Appl. Surf. Sci..

[cit37] Tsai K.-A., Hsu Y.-J. (2015). Appl. Catal., B.

[cit38] Bhattacharya S., Md Pratik S. (2021). Comput. Theor. Chem..

[cit39] Ng C. H., Ohlin C. A., Winther-Jensen B. (2015). Dyes Pigm..

[cit40] Huo J., Yu D., Li H., Luo B., Arulsamy N. (2019). RSC Adv..

[cit41] Xu Q., You H., Jia Y., Yu Y., Li H. (2021). Chemosphere.

[cit42] Shanmugam S., Ulaganathan P., Swaminathan K., Sadhasivam S., Wu Y.-R. (2017). Int. Biodeterior. Biodegrad..

[cit43] Huang Y.-R., Kong Y., Li H.-Z., Wei X.-M. (2020). Environ. Technol. Innovation.

[cit44] Abdellah M. H., Nosier S. A., El-Shazly A. H., Mubarak A. A. (2018). Alexandria Eng. J..

[cit45] de Oliveira Guidolin T., Possolli N. M., Polla M. B., Wermuth T. B., Franco de Oliveira T., Eller S., Klegues Montedo O. R., Arcaro S., Cechinel M. A. P. (2021). J. Cleaner Prod..

