# Supplementary material for: Is degradation of dyes even possible without using photocatalysts? – a detailed comparative study
Source: RSC Adv. 2022 Nov 30;12(53):34335–45. doi: 10.1039/d2ra05779d (PMC9709591; doi:10.1039/d2ra05779d)
Supplement: RA-012-D2RA05779D-s001 [file RA-012-D2RA05779D-s001.pdf]

Electronic Supplementary Material (ESI) for RSC Advances.
This journal is © The Royal Society of Chemistry 2022

Supporting file for

Is Degradation of Dyes Even Possible Without Using 'Photocatalyst'? – A Detailed Comparative Study

Subhadeep Sen[a], Chanchal Das[a], Narendranath Ghosh[b], Nabojyoti Baildya[c], Sumantra Bhattacharya[d], Moonis Ali Khan[e], Mika Sillanpää[f,g], Goutam Biswas[a]*

[a]*Department of Chemistry, Cooch Behar Panchanan Barma University, Cooch Behar, West Bengal, 736101, India.*

[b]*Department of Chemistry, University of Gour Banga, Mokdumpur, Malda-732103, India.*

[c]*Department of Chemistry, University of Kalyani, Kalyani 741235, India*

[d]*Department of Chemistry, National Institute of Technology Sikkim. Barfung Block, Ravangla, South Sikkim, Sikkim, PIN-737139, India.*

[e]*Chemistry Department, College of Science, King Saud University, Riyadh 11451, Saudi Arabia.*

[f]*Department of Biological and Chemical Engineering, Aarhus University, Aarhus C, Denmark*

[g]*Department of Chemical Engineering, School of Mining, Metallurgy and Chemical Engineering, University of Johannesburg, P. O. Box 17011, Doornfontein 2028, South Africa.*

*Corresponding Author: *Department of Chemistry, Cooch Behar Panchanan Barma University, Cooch Behar, West Bengal, India 736101; Email address:* goutam@cbpbu.ac.in (G. Biswas).

Table of contents:

1. Supplementary Figures……………………………………………2
2. Supplementary Tables……………………………………………3-4

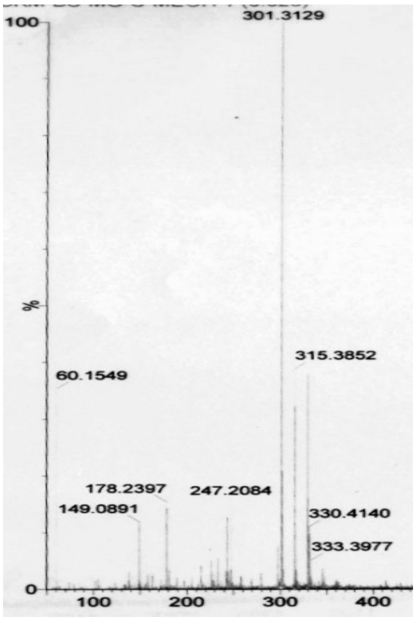

(a)

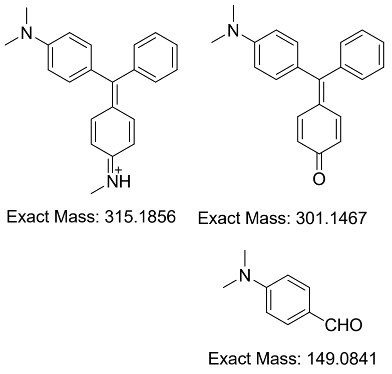

(b)

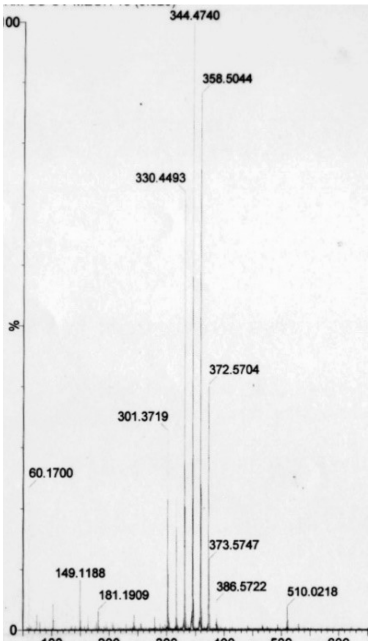

(c)

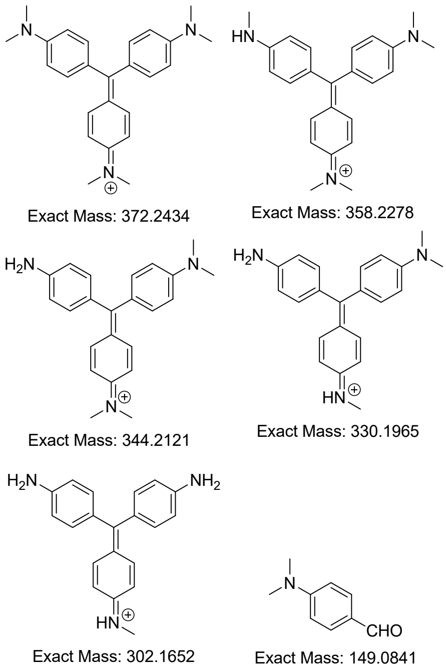

(d)

Fig. S1. Mass spectra after degradation of (a) MG and (c) CV dyes; (b) and (d) are the structures obtained after degradation of MG and CV, respectively [1–3].

Table S1

Absorption wavelengths of MG and CV in different functionals in water.

**Dye Name**	**Functional**	**Basis Set**	**λ_{max} (nm)**	**Experiment (λ_{max}, nm)**
Malachite Green	B3LYP	6-31G+(d,p)	520	617
	PBEPBE		570	
Crystal Violet	B3LYP	6-31G+(d,p)	509	590
	PBEPBE		573	

Table S2

Various properties related to absorption of MG and CV at B3LYP/6-31G+(d,p) level of theory in water.

Dye	λ_{max} (nm)	Excitation Energy (eV)	Oscillator strength	Orbital involved and % contribution
MG	570	2.1767	0.8617	H→L (100%)
	462	2.6821	0.2799	H-1→L (94%)
	408	3.0405	0.0011	H-2→L (98%)
CV	573.1	2.1632	0.6768	H-1 →L (46%), H →L (54%)
	573.0	2.1636	0.6774	H-1 →L (54%), H →L (46%)
	377	2.9353	0.0005	H-4->LUMO (94%)

Table S3

Tabulated data for electrochemical[4], computational value of the CV and MG dyes.

Dye	Cyclic Voltammetry Data			Computed Data		
	E_{ox} (eV)	E_{red} (eV)	ΔE_{CV} (eV)	HOMO (eV)	LUMO (eV)	Band Gap (eV)
CV	-5.7	-3.79	1.91	-5.72	-3.04	2.68
MG	-5.66	-4.04	1.62	-5.82	-3.29	2.53

Reference:

[1] S. Shanmugam, P. Ulaganathan, K. Swaminathan, S. Sadhasivam, Y.-R. Wu, Enhanced biodegradation and detoxification of malachite green by Trichoderma asperellum laccase: Degradation pathway and product analysis, International Biodeterioration & Biodegradation. 125 (2017) 258–268. https://doi.org/10.1016/j.ibiod.2017.08.001.

[2] Q. Xu, H. You, Y. Jia, Y. Yu, H. Li, Aquaculture drug degradation in persulfate by PANI-based microparticles controlled via ultrasonic field: Forced motion of "burning hot micromotors," Chemosphere. 275 (2021) 130098. https://doi.org/10.1016/j.chemosphere.2021.130098.

[3] Y.-R. Huang, Y. Kong, H.-Z. Li, X.-M. Wei, Removal of crystal violet by ultraviolet/persulfate: Effects, kinetics and degradation pathways, Environmental Technology & Innovation. 18 (2020) 100780. https://doi.org/10.1016/j.eti.2020.100780.

[4] C.H. Ng, C.A. Ohlin, B. Winther-Jensen, Characterisation of a series of triarylmethane dyes as light harvesters for photo-electrochemical systems, Dyes and Pigments. 115 (2015) 96–101. https://doi.org/10.1016/j.dyepig.2014.12.016.
